# Senso-Immunologic Prospects for Complex Regional Pain Syndrome Treatment

**DOI:** 10.3389/fimmu.2021.786511

**Published:** 2022-01-05

**Authors:** Takayuki Okumo, Yasunori Takayama, Kenta Maruyama, Mami Kato, Masataka Sunagawa

**Affiliations:** ^1^ Department of Physiology, Showa University School of Medicine, Shinagawa, Japan; ^2^ Division of Cell Signaling, National Institute for Physiological Sciences, Natural Institutes for Natural Sciences, Okazaki, Japan; ^3^ Department of Molecular and System Pharmacology, Graduate School of Pharmaceutical Sciences, Kyushu University, Fukuoka, Japan

**Keywords:** complex regional pain syndrome, TRPA1, CGRP, Sudeck atrophy, nociceptor, magnoflorine, Kampo formula, senso-immunology

## Abstract

Complex regional pain syndrome (CRPS) is a chronic pain syndrome that occurs in tissue injuries as the result of surgery, trauma, or ischemia. The clinical features of this severely painful condition include redness and swelling of the affected skin. Intriguingly, it was recently suggested that transient receptor potential ankyrin 1 (TRPA1) is involved in chronic post-ischemia pain, a CRPS model. TRPA1 is a non-selective cation channel expressed in calcitonin gene-related peptide (CGRP)-positive primary nociceptors that becomes highly activated in ischemic conditions, leading to the generation of pain. In this review, we summarize the history of TRPA1 and its involvement in pain sensation, inflammation, and CRPS. Furthermore, bone atrophy is also thought to be a characteristic clinical sign of CRPS. The altered bone microstructure of CRPS patients is thought to be caused by aggravated bone resorption *via* enhanced osteoclast differentiation and activation. Although TRPA1 could be a target for pain treatment in CRPS patients, we also discuss the paradoxical situation in this review. Nociceptor activation decreases the risk of bone destruction *via* CGRP secretion from free nerve endings. Thus, TRPA1 inhibition could cause severe bone atrophy. However, the suitable therapeutic strategy is controversial because the pathologic mechanisms of bone atrophy in CRPS are unclear. Therefore, we propose focusing on the remission of abnormal bone turnover observed in CRPS using a recently developed concept: senso-immunology.

## 1 Introduction

Complex regional pain syndrome (CRPS) is a chronic pain disease triggered by an inciting injury, such as fracture or surgery, affecting nearly 26 people per million worldwide (1). CRPS generally presents with allodynia, hyperalgesia, motor dysfunction, skin color change, abnormal skin temperature, edema, and localized bone atrophy. The disease noticeably differs from other neuropathic pain-related disorders in that there is long-lasting local inflammation and autonomic dysfunction, and the affected area does not correspond with dermatome distribution.

### 1.1 History

The first case report of a patient suspected to have CRPS occurred in the 16th century when Ambroise Pare noted that King Charles IX of France was suffering from severe arm pain after bloodletting (2). In 1864, Silas Weir Mitchell reported a similar patient in North America who complained of redness and burning pain at the site of a gunshot wound received in the Civil War (3). Mitchell coined the name causalgia for signs of severe pain that did not match the course and degree of trauma. In 1946, James A. Evans reported a patient similar to Mitchell’s who complained of pain associated with autonomic dysfunction, and he proposed the condition as reflex sympathetic dystrophy (4). In the second half of the 20th century, the disease was given various names, including Sudeck atrophy, transient osteoporosis, sympathetically maintained pain, and sympathetically independent pain. In 1994, the Task Force of the International Association for the Study of Pain unified these different names and coined the condition complex regional pain syndrome. CRPS is described as an array of painful conditions that are characterized by a continuing regional pain that is evidently disproportionate in time and degree to the usual course of any known traumatic injury or other lesion. The pain is regional, not in a specific nerve territory or dermatome, and shows progressive abnormal sensory, motor, sudomotor, and/or vasomotor dysfunction, especially at the distal extremities. CRPS is classified according to its cause as type 1 (without nerve damage, formerly known as reflex sympathetic dystrophy) or type 2 (obvious nerve damage associated with trauma or surgery, formerly known as causalgia). Approximately 90% of CRPS patients are diagnosed with type 1 (5–7).

### 1.2 Epidemiology

Various epidemiologic studies of CRPS have been conducted. The first report was by Sandroni et al. in 2003 (5), and although there were some study limitations, such as a small number of patients, it became the basis for risk factors for CRPS and its prevalence. In 2018, an epidemiologic study with a large number of subjects (7) reported similar results to those of Sandroni et al. (5). The male:female ratio of the disease was 29%:71% (7). Additionally, the upper limbs were affected in 70% of the patients, and approximately 90% were diagnosed with CRPS type 1 (7). De Mos et al. (1) reported a similar result, showing that postmenopausal women were most likely to develop CRPS.

CRPS can be triggered by fractures of the upper limbs, especially in the forearm (8). When CRPS develops in the lower extremities, trauma to the ankles or intra-articular injury have a high risk of initiating CRPS (9). Regarding comorbidities, musculoskeletal diseases, such as rheumatoid arthritis, were reported to increase the risk of developing CRPS. High-energy trauma, severe fractures, and a prolonged time under general anesthesia during surgery are also associated with CRPS development (10).

## 2 Clinical Summary of CRPS

### 2.1 Clinical Manifestation

CRPS patients present with various symptoms, including severe pain in the affected limb, sensory disturbance, autonomic dysfunction, motor malfunction, skin atrophy with a hot or cold temperature, and localized bone atrophy. The characteristic symptom in the early stage of the disease is an intense burning or throbbing pain in the affected limb (11) that is not commensurate with the time and degree of the ordinary course of any traumatic injury. CRPS develops around the injured area 4–6 weeks after the inciting event (12) and then spreads in a distribution that does not correspond to a dermatome (8, 13).

**Figure 1:**
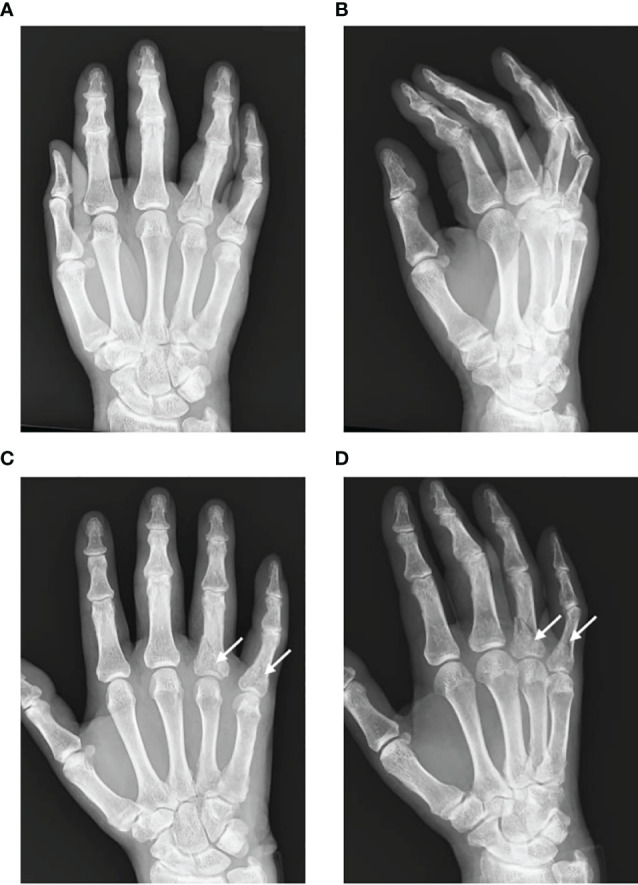
Referential X-ray images in a patient with CRPS following phalanx fractures. The patient underwent surgical treatment for fractures of the right fourth and fifth proximal phalanges and was diagnosed with CRPS due to persistent throbbing pain, swelling of the fingers, heat sensation, abnormal sweating in the palm, and a limited range of motion of the fourth and fifth fingers. **(A, B)** Radiographic images at the time of the injury. **(C, D)** Radiographic images at 5 months postoperatively. White arrows indicate localized patchy bone atrophy, which is not commensurate with the ordinary course of fracture healing.

CRPS symptoms are exacerbated by movement, contact, environmental temperature changes, and psychological stress and sometimes become intense at night (7, 12). Motor dysfunction is prominently associated with severe pain, and approximately two-thirds of patients with CRPS experience difficulty gripping with their hands or standing on their tiptoes (7, 11, 12). Autonomic dysfunction manifests as changes in skin temperature and color, sweating, edema, nail and hair atrophy, and subcutaneous bleeding. The most common symptom is skin color change and edema, occurring in over 70% of CRPS patients. As the disease progresses, hair growth slows, and the nails become brittle. Skin temperature may not only increase but also decrease, resulting in the extremities feeling cold (13). Localized bone atrophy is sometimes observed early in the disease and becomes apparent as it progresses (**Figure 1**) (12).

### 2.2 Pathophysiology

Although the precise mechanism of CRPS development remains unelucidated, the most widely accepted etiology is a combination of various factors, such as tissue inflammation, the neuroimmunologic response, sensitization of the nociceptive nervous system, and autonomic dysfunction, which are triggered by the inciting trauma. It has also been suggested that genetic tendencies and psychological state influence CRPS development.

#### 2.2.1 Inflammation and Neuroimmunology

An immune response is considered the basis for CRPS development (**Table 1**). When subjected to substantial nociceptive stimuli, such as trauma or surgery, action potentials are generated in the nociceptive neurons, and at the same time, neuropeptides, such as substance P and calcitonin gene-related peptides (CGRPs), are released around the injured tissue (24). Next, immune cells, including mast cells, are attracted around the damaged tissue (14). The neuroimmunologic response to noxious stimuli enhances the release of inflammatory mediators, such as tumor necrosis factor-α (TNF-α), interleukin-1β (IL-1β), interleukin-6 (IL-6), prostaglandin E2 (PGE2), and nerve growth factor (NGF), lowering the threshold for neuronal depolarization (10, 14, 15), which establishes peripheral nerve sensitization. Additionally, cytokines and neuropeptides released from immune cells increase vascular tissue permeability, resulting in clinical findings such as edema and skin warmth (17). Furthermore, autoimmune system may be involved in the pathophysiology of CRPS. Kohr et al. reported that the autoantibody against β2-adrenergic receptors and m2-acetylcholine receptors were observed in CRPS patients (22, 23). Other studies have shown that topical administration of serum-derived IgG from CRPS patients causes mechanical allodynia and increases tissue substance P (19, 20). It has been suggested that the immune response in CRPS patients produces autoantibodies against autonomic nerves or sensory nerves and contributes to allodynia or hypersensitivity in the injured region (19, 20).

**Table 1:** Relevant information regarding immunological mechanism of CRPS.

Inciting event	Immunologic relevance to CRPS		Etiology of CRPS
Fracture Surgery Bone bruise Burn injury etc.	Neuroinflammation TNF-α, IL-1β, NLRP3, IL-6, Substance P, CGRP Prostaglandin E2, NGF		Facilitating the depolarization of afferent neurons (10, 14, 15)
			Localized bone resorption by activated osteoclast (16)
			Increased vascular permeability (17) dysregulated vascular vasoconstriction (18) Prolonged aggravation of neuroinflammation (19–21)
	Autoimmune response		
	Autoantibodies	β2-adrenergic receptors	
		m2-acetylcholine receptors	Autonomic dysfunction (22, 23)

TNF-α, tumor necrosis factor-α; IL-1β, interleukin-1β; NLRP3, NLR family pyrin domain containing 3; IL-6, interleukin-6; CGRP, calcitonin gene-related peptides; NGF, nerve growth factor.

#### 2.2.2 Interaction Between Peripheral Nerve Sensitization and Autonomic Dysregulation

Interaction between nociceptive neurons and the sympathetic nervous system in CRPS patients has been suggested because pain is intensified following sympathetic nerve stimulation (9). This interaction leads to hyperalgesia from sustained sympathetic firing (25) due to the increased expression of α1 adrenergic receptors and enhanced susceptibility to catecholamines in the peripheral nociceptive neurons (26, 27). Additionally, changes in serum norepinephrine concentration regulate the degree of vasoconstriction, resulting in various skin conditions in CRPS patients. In the acute phase, norepinephrine decreases vasoconstriction, resulting in edema and increased skin temperature. In the chronic phase, catecholamine sensitivity increases over time, resulting in excessive vasoconstriction, excessive sweating, and a cold sensation in the extremities (28). Thus, some researchers have categorized CRPS as hot and cold subtypes (18).

#### 2.2.3 Central Nervous System Sensitization

The continual firing of peripheral nociceptive neurons enhances the efficiency of the synaptic transmission of nociceptive stimuli in the dorsal horn of the spinal cord (29). Neuropeptides, such as glutamate and substance P, lower the threshold for mechanical and thermal stimuli, causing central sensitization, which leads to hyperalgesia and allodynia. In the chronic phase of CRPS, structural changes in the central nervous system may be observed, such as atrophied gray matter in the somatosensory area on the side of the affected limb compared with the contralateral healthy side (30). It was suggested that due to central sensitization, the longer the patient suffers from CRPS, the more painful the condition becomes. Furthermore, motor malfunction, such as a limited range of motion in the distal limbs and dystonia, are observed (31). It was also reported that the intrathecal administration of gamma-aminobutyric acid was clinically effective (32), suggesting that structural changes and alterations in motor function and the somatosensory system occur in CRPS patients.

#### 2.2.4 Genetic Influence

The association of specific genotypes with CRPS has been explored, and one case-control study reported that human leukocyte antigen subtype DQ1 was highly expressed in patients with CRPS type 1 (33). Further detailed studies revealed that the gene expression levels of human leukocyte antigen-A29.1, matrix metalloprotease 9, alanine aminopeptidase N, L-histidine decarboxylase, granulocyte colony-stimulating factor 3 receptor, and signal transducer and activator of transcription 3 were higher in CRPS patients than in healthy individuals (34).

#### 2.2.5 Psychological Stress

It is believed that disruption of the psychological stress response triggers the CRPS development. Patients who already have post-traumatic stress disorder are more likely to develop CRPS (35). Severe anxiety, helplessness, and fear of pain tend to exacerbate the course of the disease. These psychological stress responses lead to increased catecholamine secretion, a lower threshold for noxious stimuli, and sympathetic nerve overdrive. Pain catastrophizing affects CRPS development profoundly (36), and patients with such psychological tendencies have persistently higher pain scores (37).

### 2.3 Diagnosis for CRPS

#### 2.3.1 Diagnostic Criteria

There is no “gold standard” test to diagnose CRPS, and clinical diagnosis is made by identifying signs of the disease from the patient’s medical history and performing a physical examination. The first diagnostic criteria were presented in 1994 by the International Association for the Study of Pain (38). However, this diagnostic tool had low specificity and was prone to misdiagnosis. A new diagnostic tool, named the Budapest criteria, was established in 2003, and is now in mainstream use (**Table 2**) (39). The Budapest criteria show high sensitivity and specificity in differentiating CRPS from other chronic pain disorders (7).

**Table 2:** Budapest criteria for the clinical diagnosis of CRPS (37).

Criteria	
1	Continuing pain, which is disproportionate to any inciting event
2	Must report at least one symptom in three of the four following categories:
	Sensory: Reports of hyperalgesia and/or allodynia
	Vasomotor: Reports of temperature asymmetry and/or skin color changes and/or skin color asymmetry
	Sudomotor/edema: Reports of edema and/or sweating changes and/or sweating asymmetry
	Motor/trophic: Reports of decreased range of motion and/or motor dysfunction (weakness, tremor, dystonia) and/or trophic changes (hair, nail, skin)
3	Must display at least one sign at the time of evaluation in two or more of the following categories:
	Sensory: Evidence of hyperalgesia (to a pinprick) and/or allodynia (to light touch and/or deep somatic pressure and/or joint movement)
	Vasomotor: Evidence of temperature asymmetry and/or skin color changes and/or asymmetry
	Sudomotor/edema: Evidence of edema and/or sweating changes and/or sweating asymmetry
	Motor/trophic: Evidence of decreased range of motion and/or motor dysfunction (weakness, tremor, dystonia) and/or trophic changes (hair, nail, skin)
4	There is no other diagnosis that better explains the signs and symptoms

#### 2.3.2 Other Diagnostic Tools

Various examination techniques are used to assist in CRPS diagnosis. Thermography is commonly used, and changes in skin temperature are one of the CRPS severity score criteria (40). A difference in the skin temperature of the affected area of ≥1 °C compared with other areas is considered remarkable. However, large temperature differences do not appear to correlate with the perceived degree of pain (41). Bone scintigraphy is another tool used to diagnose CRPS, but its usefulness is limited due to its high specificity but low sensitivity in evaluating CRPS type 1 (42). One meta-analysis concluded that it was a useful method to confirm CRPS but an unsuitable tool for differential diagnosis (43). Radiographic imaging, such as plain X-ray radiography or computed tomography, can reveal patchy bone osteoarthritis or a Swiss cheese-like appearance in CRPS patients (16), but these features cannot be useful for diagnosis. Further research to develop a method that can clearly diagnose CRPS is crucial.

### 2.4 Treatment

The current therapeutic strategy for CRPS is a combination of physiotherapy, occupational therapy, psychotherapy, neuropathic pain medication, anti-inflammatory agents, and interventional treatment (12, 44, 45). Neuromodulation therapy, such as spinal cord stimulation (SCS) and dorsal root ganglion (DRG) stimulation, are also mentioned below.

#### 2.4.1 Physical/Occupational/Psychological Therapy

CRPS treatment guidelines recommend a multifaceted approach that includes physical therapy, occupational therapy, and psychotherapy (37). Due to the severe pain in CRPS, patients tend to avoid using the affected limb, which often impairs its motor function. The goal of rehabilitation therapy is to improve the motor function and range of motion of the affected limb and to reduce severe pain (37). A Cochrane review investigating 18 randomized controlled trials (RCTs) based on rehabilitation therapy reported that most trials were of low quality with high-risk bias. However, two treatments, namely, graded motor imagery and mirror therapy, were found to improve function and pain in patients with CRPS type 1 (46).

Graded motor imagery proceeds in three stages. In the first stage, the patient recognizes the left and right limbs by looking at pictures. In the second stage, the patient imagines the movement of the limb position shown in the pictures. In the third stage, the patient observes the limb position and movement of the healthy limb reflected in a mirror to give the illusion of moving the affected limb (47). A graded motor imagery trial showed that improvements in pain and function in patients with CRPS type 1 were maintained 6 months after the therapy (48). Mirror therapy was initially reported as a treatment for phantom limb pain (49) but has also been used to treat CRPS (50). First, the patient imagines the movements of both limbs and distinguishes between the affected side and the healthy side. Then, a mirror is placed between the healthy limb and the affected limb so that the reflection of the movement of the healthy limb in the mirror gives the illusion of moving the affected limb.

CRPS pain induces catastrophic thinking, and patients avoid using the affected limb. Therefore, treatments that assist in coping with pain through psychotherapy, such as relaxation training, thermal biofeedback, and graded exposure therapy, are also effective (37). Physical, occupational, and psychological therapy improve function, mobility, quality of life, and pain control. These treatments are the first choice for many physicians.

#### 2.4.2 Neuropathic Pain Medications

Neuropathic pain medication is used to improve the neurologic symptoms of CRPS (16). Gabapentin, which binds to the α2δ subsystem of potential-dependent calcium channels (51, 52), the tricyclic antidepressant amitriptyline (53), and the anticonvulsant carbamazepine (54) have been studied, but evidence of their usefulness as therapeutic agents is minimal. At present, the use and selection of neuropathic pain medication depend on the preference and experience of the physician.

#### 2.4.3 Anti-Inflammatory Medications

Although local inflammation or neuroinflammation is associated with CRPS onset and chronicity in the early stage of CRPS, the use of nonsteroidal anti-inflammatory drugs (NSAIDs) to reduce CRPS symptoms is ineffective (54). NSAIDs inhibit cyclooxygenase-1 and -2 and suppress the production of prostaglandins that cause inflammation. The short-term efficacy of the cyclooxygenase-2 selective inhibitor parecoxib was investigated for CRPS-induced pain and edema, but there was no difference in clinical outcomes compared with the placebo group (55). One RCT compared the effects of the corticosteroid prednisolone (40 mg/day) and the NSAID piroxicam (20 mg/day) on post-stroke CRPS type 1 patients (56). After 1 month of treatment, the prednisolone group showed a greater improvement in CRPS symptoms than the piroxicam group (56). This suggests that although NSAIDs inhibit cyclooxygenase, the effect may be insufficient to alleviate inflammation in CRPS. There are various reports showing that corticosteroid administration is effective against CRPS (57–59). On the other hand, a small double-blind, randomized study investigating the analgesic effect of a single intrathecal dose of methylprednisolone (60 mg) in patients with chronic CRPS was found to be ineffective in an interim presentation, so the study was discontinued (60). The ineffectiveness of the intrathecal administration of corticosteroids in the study might have been due to the selection of subjects with severe, chronic CRPS (60). Furthermore, there are various concerns of adverse events accompanying the long-term use of corticosteroids so that short-term corticosteroid use may be safe and effective for patients with early-stage CRPS.

#### 2.4.4 Interventional Treatment Options

##### 2.4.4.1 Sympathetic Nerve Block

A sympathetic nerve block is commonly used to treat patients with CRPS. A lumbar sympathetic nerve block is performed in patients with CRPS in the lower limbs, and a stellate ganglion block is performed in patients with CRPS in the upper limbs. However, the conclusion of a Cochrane review of the effectiveness of a sympathetic block for CRPS was controversial because it was determined neither effective nor ineffective due to the overall low level of evidence (61). Furthermore, no difference in clinical outcome was observed in two RCTs comparing a sympathetic nerve block with a placebo.

##### 2.4.4.2 Spinal Cord Stimulation

SCS is an interventional technique that electrically stimulates the dorsal horn of the spinal cord (62). An electrode is percutaneously passed through the epidural space and connected to a stimulator, which is then subcutaneously implanted in patients who report an analgesic effect due to electrode stimulation. Electrical stimulation of the dorsal horn of the spinal cord is thought to reduce excessive firing of the spinal cord neurons by activating the gamma-aminobutyric acid-transmitting pathway. A systematic review of the effects of SCS on patients with CRPS rated it as providing a high level of evidence of pain relief and improved quality of life (62). Because the placement of the stimulating electrode in an appropriate position in the epidural space is technically challenging in neuromodulation techniques, such as SCS, it is recommended that implantation should be performed at a specialized facility (63).

##### 2.4.4.3 DRG Stimulation

DRG stimulation is a relatively new technology that followed SCS. It electrically stimulates the DRG, which is an aggregate of afferent nerve cell bodies. A series of cases suggested the efficacy of DRG stimulation in CRPS patients (64). The ACCURATE study (a multicenter, prospective randomized trial) was conducted in 152 patients with CRPS in the lower extremities who were randomly assigned to either SCS or DRG stimulation groups. Higher analgesic effects were observed in the DRG group than in the SCS group at both 3 and 12 months. There was no difference in adverse events between the two groups (65). It was concluded that DRG stimulation could be an effective treatment for CRPS because it specifically stimulates the DRG in the painful area, and the adverse events were comparable to SCS (66).

## 3 Understanding Pathophysiology of CRPS

### 3.1 Transient Receptor Potential Ankyrin 1 (TRPA1) Activation in Ischemia

TRPA1 is a non-selective cation channel that belongs to the TRP superfamily (67, 68) and comprises four subunits (69). It is well-known that TRPA1 can be activated by many agonists, including allyl isothiocyanate (AITC), cold temperature, and mechanical stimuli (70). There could be more unknown agonists for TRPA1 because its activation mechanism is covalent modification of the N-terminus (71). The main activation sites in mouse TRPA1 are the C415, C422, and C622 residues (72). Additionally, TRPA1 is involved in cellular activity downstream of some receptor-type membrane proteins, including bradykinin receptor (73, 74), thymic stromal lymphopoietin receptor (75), Mas-related G protein-coupled receptors A3 and C11 (76), toll-like receptor (TLR) 4 (77), TLR7 (78), and P2X receptor and dectin-1 (79). These receptors are highly expressed in primary sensory neurons, suggesting that TRPA1 possesses unique properties that are associated with its involvement in the sensory system. For example, TRPA1 activity in primary sensory neurons induces both pain and non-histaminergic itch sensations (80). Therefore, TRPA1 should be considered a primary target to suppress the negative emotions induced by these sensations.

Importantly, TRPA1 activity is one of the plausible molecular mechanisms to explain the abnormal sensations during and after hypoxia induced by ischemia, which leads to the release of hydrogen peroxide (H_2_O_2_) and nitric oxide (NO) into the tissue. These endogenous substances are generated during ischemia, and the released H_2_O_2_ activates TRPA1 (81–83). Although NO alone does not activate TRPA1, nitroxyl (HNO, the product of NO and hydrogen sulfide), which is also generated in ischemia (84), activates TRPA1 (85).

The abovementioned phenomena occur in various tissues. For instance, pain-related behavior and licking were observed in mice by ligating the hindlimb to induce transient ischemia and after reperfusion by ligature release (86). The sustained licking time was proportional to the time of the induced ischemia. Notably, this behavior was significantly reduced in TRPA1-deficient mice but not in TRP vanilloid 1 (TRPV1)-deficient mice and was suppressed by the intraperitoneal administration of HC-030031, a selective TRPA1 antagonist. Thus, TRPA1 activation in ischemia and reperfusion induces irritation *in vivo*.

TRPA1 is also involved in the development of inflammatory responses, such as vasodilation. When TRPA1 is artificially activated by cinnamaldehyde, a natural TRPA1 agonist, the primary sensory nerve releases CGRP and NO to vascular smooth muscle cells. This leads to the relaxation of vascular smooth muscle cells *via* ATP-sensitive potassium channel activation (87). Upstream of this TRPA1-CGRP/NO pathway, HNO release from endothelial cells is a critical mechanism that activates TRPA1 (85). These facts suggest TRPA1 as a potential principal target to reduce ischemia-induced inflammation and pain.

### 3.2 TRPA1 Involvement in CRPS

TRPA1 is expressed in many tissues, and its involvement in some diseases such as respiratory disorders and visceral hypersensitivity has been reported (70). Although the entire mechanism has not been thoroughly investigated, the pain sensation in CRPS could be mainly induced by TRPA1 activation. In a CRPS-like rat model, chronic post-ischemia pain (CPIP) was observed in areas with severe ischemic damage (88). The hind paws of the rats were tightly bound by a tourniquet and reperfused a few hours later. This led to severe edema due to an accelerated increase in vascular permeability a few hours after reperfusion (88), and allodynia continued for over a month in approximately 70% of the rats. Therefore, the occurrence of these events within a few hours after reperfusion suggests that this process is a crucial mechanism in the induction of CRPS-like conditions.

The involvement of the capsaicin receptor TRPV1 in CPIP has been reported, although the mechanism of TRPV1 activation remains unclear (89). Meanwhile, TRPA1 involvement in CPIP has also been suggested. CPIP-induced mechanical and cold allodynia was reduced by the intraperitoneal administration of HC-030031 (100 mg/kg) in male Wistar rats (90). Notably, HC-030031 inhibited allodynia in both the acute and chronic phases of CPIP. Furthermore, no differences in the sexual distribution of CPIP-induced allodynia were reported in mice, and the pain-related behavior was almost completely inhibited in TRPA1-deficient mice (91, 92). The level of oxidative stress markers was reported to increase in the tibial nerve of CPIP mice. Intriguingly, this phenomenon noticeably vanished in Schwann cell-specific TRPA1-deficient mice. These facts correspond with a previous report indicating that TRPA1 expression in Schwann cells plays a crucial role in causing allodynia under conditions of oxidative stress induced by partial sciatic nerve ligation (93). Thus, the administration of a TRPA1 inhibitor is recommended as a symptomatic therapy for CRPS. Although the pain reduction by TRPA1 antagonists or antioxidants only lasted a few hours (92), this experimental evidence could give new hope to CRPS patients.

### 3.3 Is the Fundamental Cause of CRPS in the Spinal Cord?

The administration of TRPA1 inhibitors could be a prospective strategy to reduce CRPS-associated pain. But does it cure the disease itself? Although we cannot currently answer this question, we would like to discuss this further.

The pathologic events in the spinal cord that are observed in CPIP, and partial sciatic nerve ligation pain models are similar. Usually, the intracellular chloride concentration is maintained at a fairly low level by the dephosphorylation of threonine residues in potassium-chloride cotransporter 2 (KCC2) dimers but not by the dephosphorylation of tyrosine residues in KCC2 monomers (94, 95). In a peripheral nerve injury model, microglia activity increased ipsilaterally in the spinal cord, followed by the release of brain-derived neurotrophic factor from microglia (96, 97). Brain-derived neurotrophic factor (BDNF) decreases KCC2 expression through cAMP response element-binding protein activity in central nervous system neurons (98, 99). Therefore, the intracellular chloride concentration increases, and inhibitory input that depends on gamma-aminobutyric acid (GABA) or glycine changes to excitatory input (100). Thus, the neuronal circuit inhibiting the pain pathway in the spinal cord becomes an accelerator of nociception. The inhibitory circuit activity should be dependent on non-nociceptive stimuli, including touch sensation (101–103). However, the excitability of the neural circuits for touch sensitivity is enhanced by parvalbumin-positive inhibitory interneurons in peripheral nerve injury (104). Importantly, in a neuropathic pain model, increases in microglia activity were induced by ATP released from spinal dorsal horn neurons but not from primary sensory neurons (105). Additionally, microglia activation was reportedly enhanced in the spinal cord of CRPS patients (106). This phenomenon is similar in rats with CPIP (107). Collectively, this evidence indicates that the central nervous system is a valid target to cure CRPS.

### 3.4 NLR Family Pyrin Domain Containing 3 (NLRP3) Inflammasome Involvement in CRPS

The activities of glial cells in the spinal cord could be essential mechanisms involved in chronic pain induction. Additionally, inflammation is also exacerbated in pathologic spinal cord environments. An investigation of the gene expression pattern in the spinal cord of CPIP rats by RNA-sequencing and western blot suggests the upregulation of NLRP3 inflammasomes (21). NLRP3 expression is enhanced downstream of TLR4, TNF receptor, and IL-1 receptor (108). Its inflammasome is formed by NLRP3, apoptosis-associated speck-like protein containing a caspase-recruitment domain, and pro-caspase-1 (109). Caspase-1 matures in this huge protein complex and activated caspase-1 causes pyroptosis. In this molecular mechanism, the NLRP3 activity is modulated by dopamine. There are two types of dopamine receptors, excitatory and inhibitory. Dopamine receptor D1 and D5 are Gs protein-couple receptor, and D2, D3 and D4 are Gi protein-couple receptor. cAMP is metabolized in downstream of adenylyl cyclase activation, and NLRP3 protein is ubiquitinated by MARCH7, an E3 ubiquitin ligase. The ubiquitinated NLRP3 is removed by autophagy (110). The ubiquitination of NLRP3 is accelerated by cAMP increase (111). According to previous report, dopamine inhibits NLRP3 activity *via* dopamine receptor D1, but not D2, in bone marrow-derived macrophages (110). Intriguingly, electroacupuncture at the sciatic nerve of mice enhances dopamine release from the adrenal medulla, followed by a whole-body anti-inflammatory response (112). The dopamine should be released from adrenal gland *via* prokineticin receptor 2 (PROKR2)-positive DRG neurons and dorsal motor nuclei of the vagus (DMV) pathway in the electroacupuncture (113). Moreover, pain reduction was observed in CPIP rats after electroacupuncture, although the expression level of NLRP3 mRNA remained unchanged (114). These facts indicate that stimulation inducing NLRP3 autophagy is a therapeutic strategy, and electroacupuncture could be an effective treatment *via* a generalized dopamine-dependent anti-inflammatory response.

### 3.5 Negative Effect of Nociceptor Suppression in Pain

On the surface, TRPA1 inhibition appears an attractive method of pain control in CRPS. In fact, CGRPs released through primary sensory neuronal excitation protect bones against destruction (115, 116). This CGRP-dependent molecular mechanism was clearly demonstrated in mice infected with *Candida albicans* on their hind paws. These mice showed acute pain-related behavior because β-glucan, which is a component of the cell wall of *Candida albicans*, directly and indirectly stimulates TRPA1-positive primary sensory neurons (79). In this case, severe bone resorption occurred in the calcaneus of Nav1.8-positive neuron-deficient mice, TRPV1- and/or TRPA1-deficient mice, and mice that had been administered resiniferatoxin (79). Further investigation clarified that CGRPs released from primary sensory neurons suppressed osteoclast fusion, leading to bone resorption (79). Thus, bones could be concomitantly protected by some drugs used for pain control in CRPS.

## 4 Bone Involvement in CRPS

As mentioned above, CRPS is triggered by traumatic injuries, such as fractures of the extremities or surgery involving the bone. Thus, an abnormal pathologic condition in bone appears to be strongly related to CRPS onset (117). Epidemiologically, the high prevalence of CRPS in postmenopausal women (1) indicates that abnormal bone metabolism is associated with the pathology of the disease. For this reason, it was recently suggested that alteration of the bone microenvironment might be involved in the onset and early stage of CRPS (118, 119). We propose that favorably regulating the bone metabolism after trauma is critical to suppress the onset and progression of CRPS and suggest that bone-targeting therapeutic approaches should be effective.

### 4.1 Bone-Targeting Treatment Options for CRPS

#### 4.1.1 Bisphosphonates

Bisphosphonates are widely used to treat osteoporosis because they suppress bone resorption by osteoclasts. Thus, they have been suggested as effective in treating CRPS patients showing abnormal uptake on bone scintigraphy (120). Although the analgesic mechanism of bisphosphonates has not been elucidated, it has been suggested to suppress acidification of the bone microenvironment and control the release of inflammatory cytokines (121, 122). One study showed that clodronate, a first-generation bisphosphonate, suppressed the effects of Vesicular nucleotide transporter (VNUT) on neurons and immune cells, although its ability to inhibit bone resorption was weak (123). It has been suggested that clodronate may be effective in treating neuropathic pain or inflammatory pain because it inhibits ATP release from presynaptic membranes and glial cells and inhibits the release of inflammatory mediators from immune cells (123). A Cochrane review reported that there was low evidence for the efficacy of bisphosphonates in patients with CRPS (124). In contrast, another meta-analysis showed that bisphosphonates were effective (122). Recently, an RCT was reported in which intramuscular injection of neridronate was effective in patients with CRPS type 1 (125). It has been suggested that the use of bisphosphonates in the early stages of CRPS may relieve pain and prevent disease progression. However, bisphosphonates may cause drug-related adverse events, such as gastrointestinal disorders by oral administration, injection-site pain, hypocalcemia, and osteonecrosis of the jaw (126). Therefore, the identification of a safe and effective drug with less risk of side effects is desirable, and we believe that such a drug can significantly contribute to the development of a treatment strategy for CRPS.

#### 4.1.2 Natural Products

##### 4.1.2.1 Kampo Formulae

In Japan, Kampo formulae are used in general clinical practice in addition to Western medicines, and some Kampo formulae have been used for patients with CRPS (127–129). Kampo formulae are extracts used in traditional Japanese medicine (Kampo medicine) that are officially approved as ethical pharmaceuticals by the Japanese Ministry of Health, Labour and Welfare. Kampo formulae comprise two or more kinds of natural crude extracts, and decoctions of their mixtures are generally administered. Therefore, they are multicomponent formulae, having several pharmacologic actions.

We would like to highlight Boiogito (BOT), a Kampo formula consisting of six crude extracts (*Sinomenium* stem, *Astragalus* root, *Atractylodes lancea* rhizome, jujube, *Glycyrrhiza*, and ginger). BOT is frequently used to treat arthritis, especially knee osteoarthritis (130). We previously clarified that BOT administration inhibited disease progression in rats with surgically induced knee osteoarthritis. Specifically, BOT suppressed subchondral bone damage and osteophyte synthesis in the knee joint (131). Furthermore, BOT administration improved pain-related locomotive dysfunction by suppressing extracellular signal-regulated kinase 1/2 phosphorylation in the spinal dorsal horn (132). Because BOT is a Kampo formula containing magnoflorine extracted from *Sinomenium* stem as the main component, it is expected to provide an anti-osteolytic effect (133) and an anti-inflammatory effect (134). Additionally, it contains analgesic components, such as liquiritin (135, 136), isoliquiritin (137), liquiritigenin (138, 139), glycyrrhizin (140, 141), and sinomenine (142). The ideal treatment strategy for CRPS is the concurrent suppression of pain and bone atrophy, and BOT provides both effects, so it may be adapted to patients with CRPS. We plan to perform animal and clinical research to clarify its effectiveness in future investigations.

##### 4.1.2.2 Magnoflorine

Magnoflorine, an alkaloid derived from plants such as *Sinomenium acutum*, has recently been reported to prevent calvarial osteolysis by decreasing the number of mature osteoclasts (133). Calvarial osteolysis can be artificially induced by titanium particle treatment in mice (143). RANK ligand-induced osteoclast differentiation is accelerated by some signaling pathways, including transforming growth factor β-activated kinase 1, a positive regulator of osteoclast differentiation (144). Magnoflorine was reported to suppress the phosphorylation of transforming growth factor β-activated kinase 1 in a calvarial osteolysis model, with osteoclast maturation dependent on the phosphorylation of mitogen-activated protein kinases, including extracellular signal-regulated kinase, c-Jun N-terminal kinase, and p38. These factors are also involved in the inflammatory pathways and inhibit inflammation by downregulating the expression of some receptors, such as TLR4 (134). Meanwhile, Jun dimerization protein 2 (Jdp2) is considered a prominent player in osteoclast differentiation because RANK ligand-induced osteoclast differentiation was almost canceled in Jdp2-deficient mice (144). Furthermore, a recent report suggested a novel cell type, called an osteomorph, which is the daughter cell resulting from osteoclast fission (145). Although the effects of magnoflorine in these events are yet to be elucidated, the reported facts suggest that it suppresses both bone atrophy and inflammatory pain in CRPS.

## 5 Senso-immunology as a Driving Force in the Understanding of CRPS

**Figure 2:**
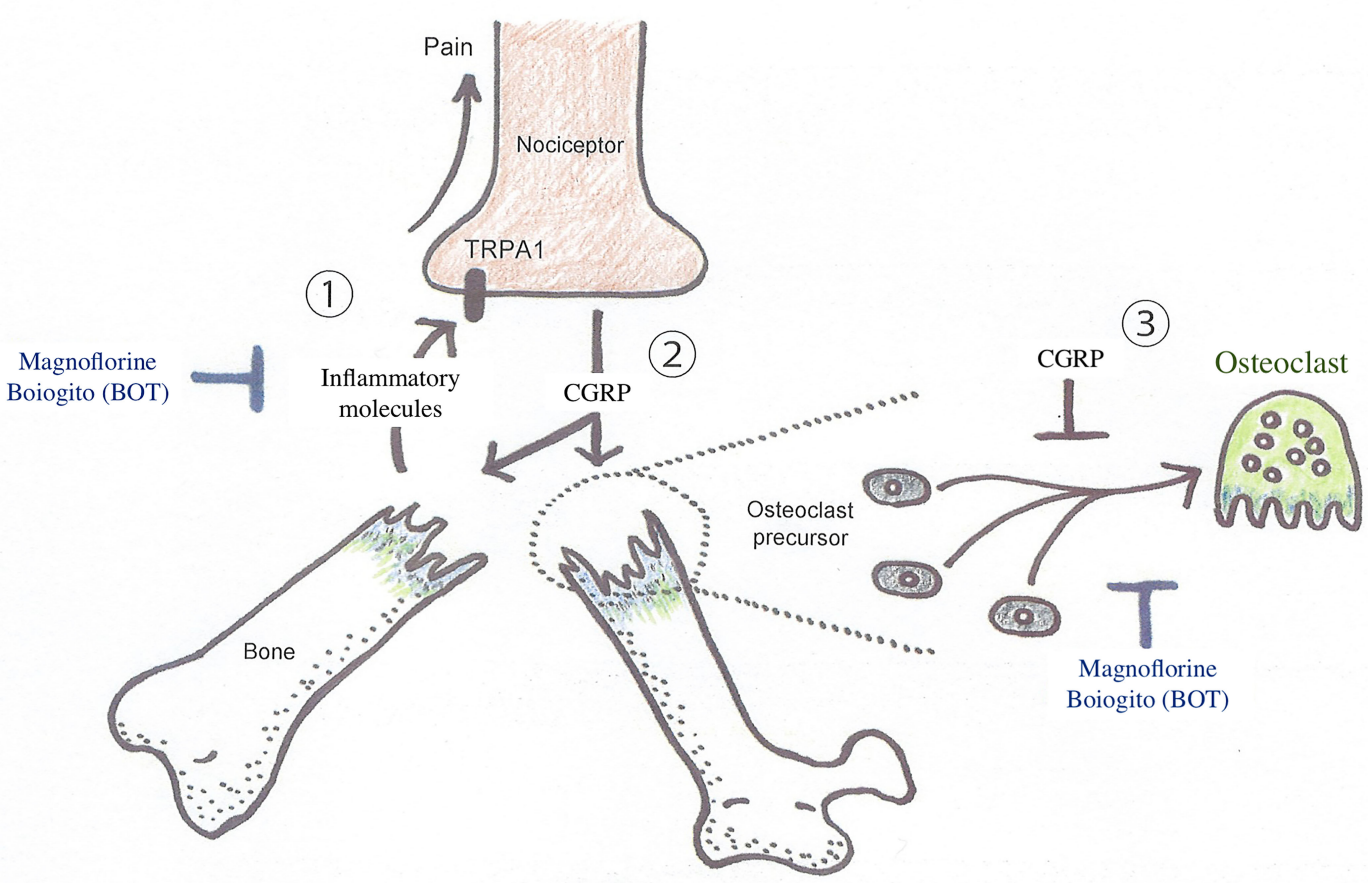
Diagrammatic illustration of the interaction between nociceptive neurons and osteoclasts in abnormal bone metabolism in CRPS triggered by trauma, such as the fracture of an extremity. A substantial injury, such as a bone fracture, produces action potentials in nociceptive neurons *via* TRPA1 activation, which transmits painful stimuli. On the other hand, osteoclast differentiation is inhibited when CGRP is released around the injured site *via* retrograde axonal transport and exocrine secretion. However, there is concern that the inflammatory response triggered by CGRP spreading around the injured tissue may lower the threshold for depolarization of many other nociceptive neurons, resulting in peripheral pain sensitization, leading to the CRPS development. Boiogito, which is rich in magnoflorine, is expected to prevent the exacerbation of symptoms and pathological conditions of early CRPS by inhibiting osteoclast differentiation and inflammation. CGRP, calcitonin gene-related peptides; TRPA1, transient receptor potential ankyrin 1.

The development of the disease concept of CRPS and its biomedical study were initiated by anesthesiologists in the late 20th century (146). However, as it became clear that inflammatory and bone metabolic mechanisms were involved in the pathogenesis of CRPS, the disease piqued the interest of orthopedic surgeons and rheumatologists. Immune cells such as lymphocytes and dendritic cells are present around nociceptors, and activated nociceptors modulate the functions of immune cells through the release of neuropeptides (147). Although nociceptors are generally known to be distributed in soft tissue, they are actually distributed within bone tissue as well, and neuropeptides derived from nociceptors affect the activity of cells that reside in the bone such as osteoclasts, osteoblasts and hematopoietic stem cells (116, 148, 149). On the other hand, cytokines and acids released from immune cells, epithelial cells, and osteoclasts can stimulate nociceptors to regulate their functions and generate pain (150, 151). Thus, it has been suggested that the immune system and the sensory system are closely related to each other to maintain our homeostasis (**Figure 2**). There are many unknowns about why CRPS occurs and how it can be cured, but this is probably due to the lack of research focusing on the crosstalk between the immune system and the sensory system. Therefore, not only clinicians but also basic immunologists and basic neurophysiologists need to collaborate in order to elucidate the pathogenesis of CRPS. We have recently coined the interdisciplinary field of studying the interaction between the immune system and the sensory system “Senso-immunology” (152). Although this new interdisciplinary concept is not widely recognized in both immunology and neurophysiology at this time, we believe that it will provide a very useful perspective for future research on CRPS.

## 6 Conclusion

Even with the accumulation of clinical research over the decades, the etiology and pathogenesis of CRPS, as well as appropriate treatment strategies, are still being explored. A severe injury, such as a fracture in an extremity, causes prolonged and exacerbated inflammation and localized abnormal bone metabolism due to the immune response triggered by the neuropeptides released from nociceptive neurons around the tissue. Thus, we suggest using the term senso-immunology to describe the immunologic interaction between nociceptive neurons and damaged tissues or bones. This review suggests that regional bone metabolic abnormalities may play an essential role in CRPS development, particularly in CRPS type 1. It would be desirable to treat patients with CRPS by suppressing excessive inflammation around the damaged tissue and improving bone metabolism, with particular emphasis on suppressing osteoclast differentiation. Although bisphosphonates have been shown to be an effective treatment for CRPS in recent years, we believe that a variety of treatment options should be offered, taking into consideration the adverse events. We propose that Oriental herbal medicines may also be effective because many medicinal plants regulate bone metabolism and possess anti-inflammatory properties, and Kampo formulae, which are the ideal combinations of these plants, might be an effective treatment for CRPS. Because there are few clinical and basic research reports on Kampo formulae, further research and accumulation of clinical evidence are necessary.

## Author Contributions

TO, YT, KM, and MK performed the literature search, designed the figures and tables, and wrote the manuscript of the review. MS performed the literature search, wrote, and supervised, the review. All authors approved the submitted version of the article.

## Conflict of Interest

The authors declare that the research was conducted in the absence of any commercial or financial relationships that could be construed as a potential conflict of interest.

## Publisher’s Note

All claims expressed in this article are solely those of the authors and do not necessarily represent those of their affiliated organizations, or those of the publisher, the editors and the reviewers. Any product that may be evaluated in this article, or claim that may be made by its manufacturer, is not guaranteed or endorsed by the publisher.
